# Mental health risks among nurses under abusive supervision: the moderating roles of job role ambiguity and patients’ lack of reciprocity

**DOI:** 10.1186/s13033-015-0014-x

**Published:** 2015-05-29

**Authors:** Jing Qian, Haiwan Wang, Zhuo Rachel Han, Jun Wang, Hui Wang

**Affiliations:** Business School, Beijing Normal University, Beijing, 100875 China; Beijing Key Laboratory of Applied Experimental Psychology, School of Psychology, Beijing Normal University, Beijing, 100875 China

**Keywords:** Mental health, Abusive supervision, Role ambiguity, Patient reciprocity

## Abstract

**Background:**

While the nursing profession has been associated with mental health problems and the research into the antecedents of mental health has steadily grown, the relationship between abusive supervision and mental health issues of anxiety and depression remains largely unknown.

**Aim:**

This study aims to examine the relationship between abusive supervision and mental health problems. And we also aim to investigate whether this relationship is moderated by role ambiguity and the patients’ lack of reciprocity.

**Methods:**

A total of 227 frontline nurses from two public hospitals completed the survey questionnaire.

**Results:**

(1) Abusive supervision was positively associated with poor mental health; (2) the positive relationship was moderated by nurses’ perceived role ambiguity in such a way that the relationship was stronger when the perceived role ambiguity is high; (3) the positive relationship was moderated by the patients’ lack of reciprocity in such a way that the relationship was stronger when patients’ lack of reciprocity was high.

**Conclusions:**

To conclude, the present study showed that abusive supervision was positively associated with mental health problems of anxiety and depression among samples of Chinese nurses. Findings of this study also highlighted that this relationship was contingent upon perceived role ambiguity and patients’ reciprocity.

## Background

The nursing profession has been associated with high workload, irregular working hours, high job demand, and emotional complexity [1, 2], and studies have suggested that nurses show more psychological and physical stress symptoms and mental health problems than individuals from other occupations [1, 3, 4]. For example, on a daily basis, nurses are often confronted with patients who do not follow their advice, make impossible demands, resist following the doctors’ instructions, and even cheat and manipulate. In addition, nurses’ mental health problems have been linked to quality of service [5, 6]. Consequently, detecting antecedents of nurses’ mental health is not only important for individual nurses, but also for patients and hospitals. Defined as “subordinates’ perceptions of the extent to which leaders engage in the sustained display of hostile verbal and nonverbal behaviors, excluding physical contact” ([7], p. 178), abusive supervision has been associated with subordinates’ mental health problems such as anxiety, depression [7], diminished self-efficacy [8], and somatic health complaints [8, 9]. According to Tepper [10], abusive supervision occurs quite often in emotional job contexts with requirements of expressing and hiding emotion such as nursing [1, 11]. The empirical evidence of abusive supervision on nurses’ mental health problems, however, has been lacking. Therefore, the first objective of the present study is to examine the relationship between abusive supervision by nurse managers and nurses’ mental health problems of anxiety and depression.

More recently, leadership researchers have adopted a contingency approach in arguing that managerial practice’s influence on subordinates may be contingent upon potential moderators such as individual differences or contextual factors (e.g., [12, 13]). Role ambiguity refers to a perceived lack of job-related information such as expectations, goals, assignments, authority, responsibilities, and other job conditions [14, 15]. Under abusive supervision, nurses may try to make the interactions with the supervision as less as possible. A poorly defined job however means that the nurses have to search job-related information from the manager nurses in order to survive and develop in the work climate. In addition, studies have shown that information from the supervisors cannot be replaced by information from other sources such as coworkers [16]. In the present study, we suggest perceived role ambiguity could make the harm of abusive supervision on nurses’ mental health problems even worse. The second objective of our study is to investigate whether the perceived role ambiguity could moderate the relationship between abusive supervision and mental health problems of anxiety and depression.

As for other health professionals, a nurse’s job is rewarding when, for example, patients show gratitude after recovery or consultation. This kind of reward has been considered to be of great significance for nurses’ meaning management, especially those under great pressure [1]. Indeed, studies have shown that patients’ showing of reciprocity to doctors is of great significance for the doctors’ psychological well being [3]. In this study, we propose that the lack of patients’ reciprocity could make the social context for nurses under abusive supervision even worse. Therefore, the third objective for us is to investigate whether the patients’ lack of reciprocity would increase the harm of abusive supervision on nurses’ mental health risks of anxiety and depression.

In the present study, we hypothesize that (1) nurse managers’ abusive supervision is positively related to nurses’ mental health problems; (2) nurses’ role ambiguity will moderate the positive relationship between abusive supervision and mental health risks in such a way that the relationship will be stronger when the role ambiguity is high; (3) patients’ lack of reciprocity will moderate the positive relationship between abusive supervision and mental health risks in such a way that the relationship will be stronger when the reciprocity from the patient is low.

## Methods

### Sample and procedure

We collected data in person from two public hospitals located in a major city in Northern China. All procedures were approved by the university’s Institutional Review Board. We invited frontline nurses to participate in the survey at monthly hospital meetings. Surveys were completed on a voluntary basis. Each packet contained an information sheet explaining the objective of the survey along with a consent form, survey questionnaire and a return envelope with seal tape to protect the respondents’ confidentiality. The respondents were instructed to complete the questionnaires, seal them in the return envelope, and put the finished questionnaires in a designated box located in the lobby. Of the 345 questionnaires distributed, 227 questionnaires were returned, representing response rates of 64.12%. Nurse respondents were predominantly female (94.7%), reported an average age of 34.03 years (SD = 7.52), an average of 12.54 years of education, and an average organizational tenure of 8.53 years (SD = 6.75).

### Measures

To ensure measure equivalence between the Chinese and English versions, the translation and back-translation method [17] was applied. With the exception of the mental health scale, we used a five-point response scale ranging from “strongly disagree” (1) to “strongly agree” (5) for all items. For mental health and lack of reciprocity, the items were scored on a five-point rating scale, ranging from (1) “never” to (5) “often.”

#### Abusive supervision

We measured abusive supervision with a 15-item scale developed by Tepper [7] which has previously been used in a Chinese context (e.g., [18, 19]). Sample items are “My supervisor tells me my thoughts or feelings are stupid” and “My supervisor expresses anger at me when he/she is mad for another reason.” The scale’s alpha reliability in this study is 0.93.

#### Mental health

Mental health was measured with six items from the Center for Epidemiologic Studies Depression Scale (e.g., “In the past few months, how often have you wondered if anything is worthwhile?”; “In the past few months, how often have you been in low spirits?”; [20]) and six anxiety items from the Diagnostic Interview Schedule (“In the past few months, how often have you felt irritable, fidgety, or tense?” and “In the past few months, how often have you felt afraid for no reason?”; [21]). Validity studies suggest that the depression and anxiety scales are good predictors of outcomes such as drug addiction and physical health (obesity, high blood pressure; see [22], for a thorough review). The reliability estimate for the scale was 0.88.

#### Role ambiguity

Role ambiguity was measured using the 5-item scale from Rizzo et al. [15]. Consistent with previous research and the conceptualization of role ambiguity, all of the items were reverse coded, which resulted in a scale for which the higher score was, the greater was the role ambiguity. A sample item stated, “I feel certain about how much authority I have.” Cronbach’s alpha for this scale was 0.70.

#### Lack of reciprocity

Lack of reciprocity in the relationships with patients was measured with a three-item scale developed by Bakker et al. [3]. A sample item is “How often do you feel you invest more in the relationship with patients than you receive in return?” The reliability estimate for the scale was 0.83.

#### Control variables

We controlled for the participants’ age, gender, education, and company tenure. Age, education, and company tenure were measured by number of years. Gender was coded 0 for “female” and 1 for “male.”

### Data analytic strategies

First, preliminary analyses evaluating the descriptive statistics and correlations among study variables, and possible group differences in study variables based on demographic characteristics were performed. Next, the two moderation models, with role ambiguity and patients’ lack of reciprocity as moderators on the relations between abusive supervision and nurses’ mental health risks were tested using SPSS MODPROBE macro, developed by Hayes and Matthes [23] for estimating the single-degree-of-freedom interactions in Ordinary Least Square (OLS) and logistic regression.

## Results

### Preliminary analyses

See Table 1 for correlations among study variables, mean scores, and standard deviations. Initial analyses examined participants’ age, gender, education, and company tenure differences on all variables. Pearson’s correlational test demonstrated that age, years of education, and years of company tenure was not significantly associated with any study variables. Independent samples t tests demonstrated no gender difference on any study variable.

**Table 1:** Means, standard deviations, and correlations among study variables

		Mean	SD	1	2	3
1	Abusive supervision	35.23	12.46			
2	Mental health risks	29.97	9.02	0.66**		
3	Role ambiguity	9.96	2.81	0.08	0.12	
4	Lack of reciprocity	17.36	4.00	0.09	−0.21**	−0.01

* *p* < 0.01, ** *p* < 0.001.

**Figure 1:**
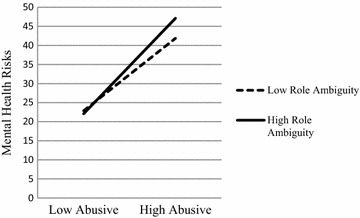
Interaction between abusive supervision and role ambiguity on mental health risks.

The first moderation analysis examined whether role ambiguity moderated the relations between abusive supervision and job burnout. Applying the modprobe macros for moderation analysis, the conditional effect of role ambiguity was estimated at values of one standard deviation below the mean, the mean, and one standard deviation above the mean. Demographic variables were again controlled in the model. Results showed that role ambiguity moderately moderated the links between abusive supervision and nurses’ mental health risks (Table 2). Specifically, as shown in Figure 1, the conditional effect estimates indicated that the interaction between abusive supervision and role ambiguity was such that abusive supervision and mental health risks was positively associated. Under the condition where there were lower levels of abusive supervision, the low role ambiguity group seemed to report more mental health risks (b = 0.40, SE = 0.06, BCa CI = 0.2826–0.5093), whereas under the condition where there were higher levels of abusive supervision, the high role ambiguity group seemed to report more mental health risks (b = 52, SE = 0.05, BCa CI = 0.4321–0.6109).

**Table 2:** Results of ordinary least square regression analyses

Moderator	b (SE)	t	R^2^	F
Patient’s lack of reciprocity (L)			0.52	81.88**
Abusive supervision (A)	0.47 (0.03)	4.85**		
Mental health risks (M)	−0.87 (0.14)	1.05**		
A × L	−0.03 (0.01)	2.81**		
Role ambiguity (R)			0.08	6.98**
Abusive supervision (A)	0.46 (0.04)	12.37**		
Mental health risks (M)	0.16 (0.11)	1.42		
A × R	0.02 (0.01)	1.74		

* *p* < 0.05, ** *p* < 0.01.

**Figure 2:**
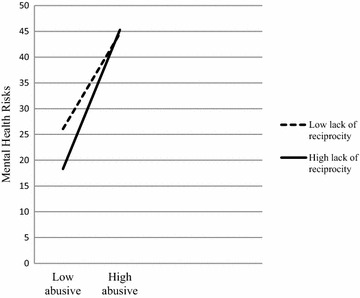
Interaction between abusive supervision and lack of reciprocity on mental health risks.

Similarly, the second moderation analysis examined whether lack of reciprocity moderated links between abusive supervision and nurses’ mental health risks. Preacher et al. [24] demonstrated that moderation is established when the independent variable and moderator significantly interact and the bootstrapped confidence interval does not contain zero. Applying the modprobe macros for moderation analysis, the conditional effect of lack of reciprocity was estimated at values of one standard deviation below the mean, the mean, and one standard deviation above the mean. Demographic variables (i.e., participants’ age, gender, years of education, and year of company tenure) were also entered in the model as controlled variables. Results showed that patients’ lack of reciprocity moderated the relations between abusive supervision and nurses’ mental health risks (Table 2). As shown in Figure 2, under the condition where there were lower levels of abusive supervision, low lack of reciprocity group had more mental health risks than higher lack of reciprocity group (b = 0.39, SE = 0.05, BCa CI = 0.2863–0.4870), but when under the condition where there were high levels of abusive supervision, high lack of reciprocity group had more mental health risks than low lack of reciprocity group (b = 0.56, SE = 0.04, BCa CI = 0.4810–0.6439).

## Discussion

While the nursing profession has been associated with mental health problems [1, 3] and the research into the antecedents of mental health risks has steadily grown, the relationship between abusive supervision and mental health issues of anxiety and depression remains largely unknown. To this end, we suggest three findings. First, abusive supervision was positively associated with poor mental health. Second, the positive relation was moderated by nurses’ perceived role ambiguity in such a way that the relationship was stronger when the role ambiguity is high. Third, the positive relationship was moderated by patients’ lack of reciprocity in such a way that the relationship was stronger when patients’ lack of reciprocity was high.

Our findings contribute to the literature on abusive supervision and mental health in several ways. First, although the implications of abusive supervision for depression and anxiety exist for the nursing occupation, they have not been empirically tested. This study is the first empirical study to integrate abusive supervision and mental health on a sample of Chinese nurses. Second, research has suggested that job roles are rarely fixed and the role perceptions can contribute to the work context, which could serve as a moderating factor for managerial behaviors and practices [25]. However little attention has been directed toward of it as a potential moderator on the influence of abusive supervision. We address this issue by suggesting that when nurses being abused by the manager (the perception about the manager), the poor defined work roles (the perception about the job context) could magnify this negative effect on mental health. Third, our findings shed new light on the role played by patients in nurses’ experience of depression and anxiety. Researchers suggest that patients play an important role in nurses’ mental health and they call for more studies to address this issue (e.g., [4, 6, 25]). In addition, to the best of our knowledge, no study has examined the moderating effect on the relationship between abusive supervision and mental health risks. In response to the call for further research into this area, we took a step forward by addressing the exploratory question of whether patients’ lack of reciprocity moderated the relationship between abusive supervision and anxiety and depression.

Turning to practical implications, nurses’ negative mental health is costly for health institutions and society [1, 3, 4]. A straightforward implication is that health institutions should pay special attention to the nurse managers’ abusive supervision in order to prevent negative mental health for nurses. For example, health organizations should make it clear that they have zero-tolerance policies for the occurrence of abusive supervision and they will deal harshly with the abusers. Previous studies have suggested that there is a trickle-down effect from abusive supervision [18]. That is, those who have been mistreated by their supervisors are more likely to abuse their subordinates. Given this, health organizations could provide certain channels such as an employee assistance program (EAP) or other support resources such as counseling and well-being workshops for all levels of workers, from top management to frontline employees, to seek help and assistance. Additional mentoring programs could also be of help here to provide social support for both supervisor nurses and subordinate nurses to seek career-related help and psychological support [26]. The second practical implication of our study stemmed from the moderators identified. After knowing that role ambiguity could strengthen the harm of abusive supervision on negative mental health, health organizations may find it helpful to clarify the job roles for nurses. Indeed, research has suggested that low role ambiguity could generate other benefits such as organizational citizenship behavior [27]. Furthermore, the moderating effect of patients’ lack of reciprocity on the relationship between abusive supervision and mental health implied that patients have their place in nurses’ mental health management, while the demonstration of their reciprocity needs to be encouraged and promoted. This can be achieved through certain organization initiated programs or platforms for patients to show gratitude to nurses such as greeting cards, thank you parties, or letters and emails.

Although this study employed a design that has been commonly applied in this area of research, it is important to recognize that some methodological refinements would strengthen the conclusions. The use of cross-sectional data implies that cause-effect relations cannot be inferred from the findings. Although our findings are consistent with the abusive supervision and mental health literature, future research should, nonetheless, adopt a longitudinal or experimental design to fully address the issue of causality. Additionally, the generalization of the results remains to be tested. The extent to which the results in the current study are applicable to other cultures is an open question since the hypotheses were only tested on Chinese nurse samples. Future studies could therefore test our model in other cultures.

## Conclusions

To conclude, the present study showed that abusive supervision was positively associated with mental health problems of anxiety and depression among samples of Chinese nurses. Findings of this study also highlighted that this relationship was contingent upon perceived role ambiguity and patients’ reciprocity.
